# Validation of Alogo Move Pro: A GPS-Based Inertial Measurement Unit for the Objective Examination of Gait and Jumping in Horses

**DOI:** 10.3390/s23094196

**Published:** 2023-04-22

**Authors:** Kévin Cédric Guyard, Stéphane Montavon, Jonathan Bertolaccini, Michel Deriaz

**Affiliations:** 1 Information Science Institute GSEM/CUI, University of Geneva, 1227 Carouge, Switzerland; jonathan.bertolaccini@unige.ch; 2 Veterinary Department of the Swiss Armed Force, 3003 Berne, Switzerland; smontavon@bluewin.ch; 3 HES-SO/HEG Genève, 1227 Carouge, Switzerland; michel.deriaz@hesge.ch

**Keywords:** horse, gait analysis, biomechanics, IMU, GPS sensor, validation

## Abstract

Quantitative information on how well a horse clears a jump has great potential to support the rider in improving the horse’s jumping performance. This study investigated the validation of a GPS-based inertial measurement unit, namely Alogo Move Pro, compared with a traditional optical motion capture system. Accuracy and precision of the three jumping characteristics of maximum height (Zmax), stride/jump length (lhorz), and mean horizontal speed (vhorz) were compared. Eleven horse–rider pairs repeated two identical jumps (an upright and an oxer fence) several times (*n* = 6 to 10) at different heights in a 20 × 60 m tent arena. The ground was a fiber sand surface. The 24 OMC (Oqus 7+, Qualisys) cameras were rigged on aluminum rails suspended 3 m above the ground. The Alogo sensor was placed in a pocket on the protective plate of the saddle girth. Reflective markers placed on and around the Alogo sensor were used to define a rigid body for kinematic analysis. The Alogo sensor data were collected and processed using the Alogo proprietary software; stride-matched OMC data were collected using Qualisys Track Manager and post-processed in Python. Residual analysis and Bland–Altman plots were performed in Python. The Alogo sensor provided measures with relative accuracy in the range of 10.5–20.7% for stride segments and 5.5–29.2% for jump segments. Regarding relative precision, we obtained values in the range of 6.3–14.5% for stride segments and 2.8–18.2% for jump segments. These accuracy differences were deemed good under field study conditions where GPS signal strength might have been suboptimal.

## 1. Introduction

Correct and tailor-made training of sport horses according to the type of equestrian discipline is of great interest as lameness is strongly linked to competition [1,2]. In this context, the structure and quality of training of sports horses are interesting to quantify and qualify. Especially show jumping, eventing, dressage, and lameness examination are areas where new technology greatly appeals [3,4]. In terms of exercise and training, every effort should be made to ensure the best possible equine welfare [5]. Inertial measurement units (IMU) are used in research and commercial products to analyze equine movement biomechanics [6,7,8,9,10]. They are typically less expensive, more versatile, and applicable under field conditions compared with optical motion capture systems (OMC) that utilize one or more cameras to capture movement. The main drawback of IMUs systems is that they do not sample the global position of the sensor but instead measure acceleration and orientation (rotation) in a local reference frame.

For many biomechanical applications such as poor performance and lameness detection, information about speed and position are crucial, and the cumulative errors caused by IMU dead reckoning can become too large of a hindrance. One situation where this applies would be during a show jumping competition where the horse has minimal linear steady-state movement. Fusing IMU sensor output with a GPS signal can alleviate this problem to some extent, but results need to be validated both technically and biomechanically against a gold-standard measurement system. The Alogo Move Pro is a single sensor IMU fitted with a GPS receiver chip. When used, it is mounted on the protective plate of the saddle girth. One of its purposes is to quantify variables related to the movement of the horse during walking, trotting, cantering, and jumping. Among other things, it calculates per stride and jump

- Maximum height (Zmax);
- Stride/jump length (lhorz);
- Mean horizontal speed (vhorz).

The Alogo algorithms fuse IMU and GPS signals to produce position and speed in a global reference frame. Furthermore, segments are extracted, classified as strides or jumps, and associated with a gait (trot, canter).

This study aimed to validate the accuracy and precision of the three measured variables of Zmax, lhorz, and vhorz against an OMC system during trotting and cantering at different speeds and jumping at different jump heights.

## 2. Materials and Methods

### 2.1. Horses and Riders

A total of 11 horse–rider pairs (HRPs) were included. One rider rode three horses while the rest rode only one. All 11 horses were Warmbloods aged between 5 and 12 years old. All horses used were deemed “fit to compete” at the time of the study. The 9 riders had different experiences in show jumping; 3 were graded as novices, 5 as intermediate, and 1 as experienced. The riders were aged 16–39, weighed 55–78 kg, and were between 155 cm and 185 cm tall. Three riders were male, and six were female.

### 2.2. Equipment

**Figure 1:**
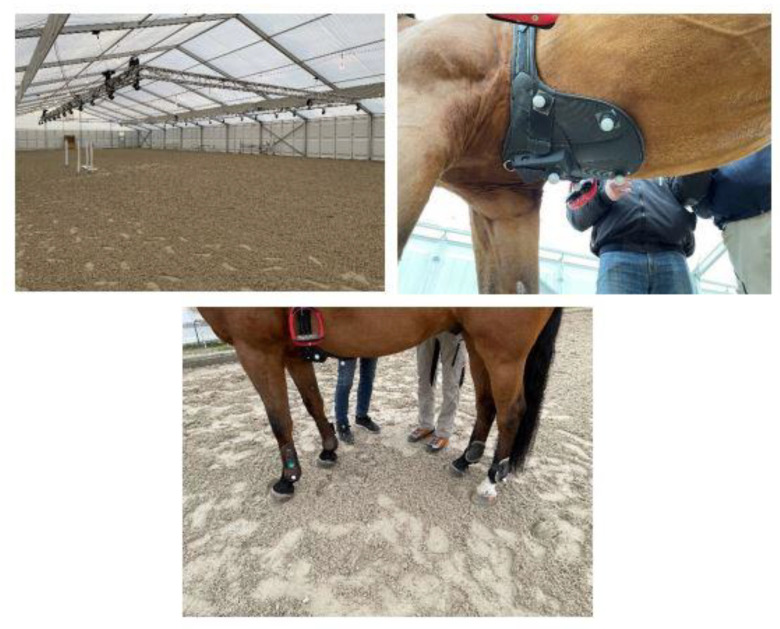
Arena, protective plate with markers, and markers on the brush boots.

**Figure 2:**
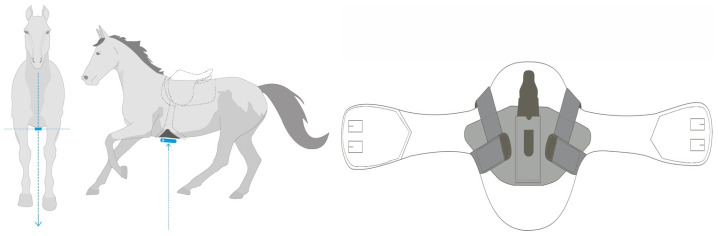
Two drawings show the positioning and mounting of the sensor.

All measurement trials were performed in a temporary tent arena 20 × 60 m (Figure 1). Before every measurement trial, each horse was fitted with the Alogo Move Pro sensor (Alogo Swiss Technology, Renens, Switzerland) and the optical markers used by the OMC system (Qualisys AB, Gothenburg, Sweden). The sensor was placed in a custom-made pocket on the protective plate on the saddle girth. It did not hinder movement and weighed only 127 g (Figure 1). Two drawings in Figure 2 show the positioning and mounting of the sensor. Reflective markers were attached using double adhesive tape on the protective plate, over to the fetlock joints on brush boots, on the saddle, on the pelvis, on ribs 15 and 16 (16 and 18 for one horse), on both sides of the belly, and the head (Figure 1). The 24 OMC (Oqus 7+) cameras were rigged on aluminum rails suspended 3 m above the floor and aimed to cover an area of 10 × 20 m in the center of the arena. OMC system sampled positions on the x, y, and z axes at 100 Hz. Alogo Move Pro sampled acceleration on the x, y, and z axes at 100 Hz. For both systems, position, and speed were derived from these measures. Based on these samples, Alogo Move Pro provided a temporal segment corresponding to the strides and jumps. On each segment, parameters Zmax, lhorz, and vhorz were computed by both systems, thus turning the temporal data comparison into a stride comparison. The temporal aspect was implicitly embedded in the stride number.

### 2.3. Experiment

Each HRP performed a short warm-up before being fitted with the measurement equipment. First, a short stance measurement was performed with the horse standing still in the middle of the OMC measurement volume. Then, the trials were performed as follows for each HRP:

- Trotting and cantering on a small radius circle on both left and right rein. At least three successful passes through the OMC measurement volume were required for each gait and left/right rein;
- Jumping single upright fences and oxers with 10 cm increments in fence height from 80–100 cm to a maximum fence height of 110–140 cm according to the horse/rider skill level. Each fence height was jumped with both the left and right approach. Jumps with a knockdown or refusal were repeated. The Alogo Move Pro sensor captured the entire trial, including stance, for each HRP, while the OMC captured measurements per jump height and fence type.

### 2.4. Data Analysis

After the acquisition, data were cleaned to address the inconsistent or missing data, and the general statistics were computed:

$$Accuracy=\frac{1}{N}*\overset{N}{\underset{n=1}{\sum }}\varepsilon \left(n\right)$$

$$Precision=\sqrt{\frac{1}{n}*\overset{N}{\underset{n=1}{\sum }}\varepsilon \left(n\right)-Accuracy}$$

CI95%=Accuracy±1.96*Precision

$$Error=\frac{1}{N}*\;\overset{N}{\underset{n=1}{\sum }}\frac{\varepsilon \left(n\right)}{x\left(n\right)}$$

where *N* is the number of samples, *x*(*n*) is the *n*th value measured by the OMC system,

- and *ε*(*n*) is the difference between the *n*th value measured by the Alogo Move Pro sensor and the *n*th value measured by the OMC system.

For every measured variable, the following plots are extracted:

- two Bland–Altman plots (one for strides and one for jumps) [11];
- three box plots of the difference between the Alogo and the OMC measures (one by HRPs, one by stride, and one by obstacle height).

To finalize, we computed the *k*-samples Anderson–Darling tests [12] on subsamples divided by HRP, stride type, and objective height to determine if there was a variation in the accuracy among these parameters.

## 3. Results

### 3.1. General Observations

As we can observe in Table 1, the Alogo Move Pro sensor provided measures with relative accuracy in the range of 10.5–20.7% for stride segments and 5.5–29.2% for jump segments. Regarding relative precision, we obtained values in the range of 6.3–14.5% for stride segments and 2.8–18.2% for jump segments.

From the *k*-samples Anderson–Darling test results in Table 2, we can conclude that the distribution of the difference in variables measured by the Alogo Move Pro sensor and the OMC system is not the same when subsampled using HRPs, stride type, or obstacle height. Thus, this highlights the fact that these three parameters have an impact on the accuracy and precision of the sensor. The impact is observed in the box plots in the three subsections below.

### 3.2. Maximum Height

**Figure 3:**
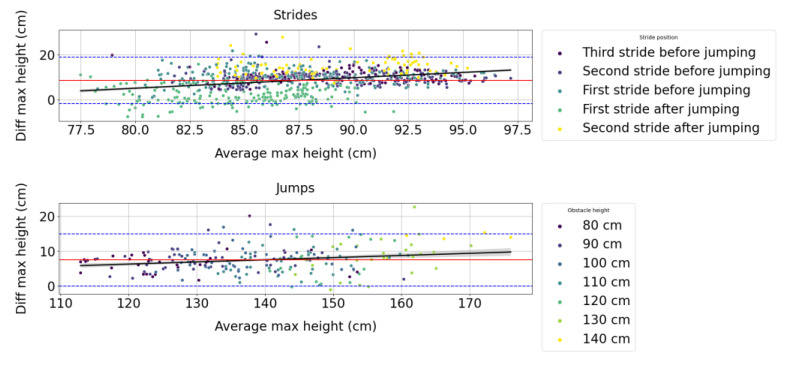
Bland–Altman plots for the maximum height (Zmax) measured by the Alogo Move Pro sensor versus the OMC system for strides and jumps. “Diff” contraction stands for difference. The red line indicates the average difference, and the blue dashed lines the 95% limits of agreement. The black line indicates the ordinary least square regression and its 95% confidence interval obtained by a 1000 bootstrap resample.

Thanks to Figure 3, we can observe that the difference in measures between the Alogo Move Pro sensor and OMC system for the maximum height variable (Zmax) follows a positive correlation with the true value.

**Figure 4:**
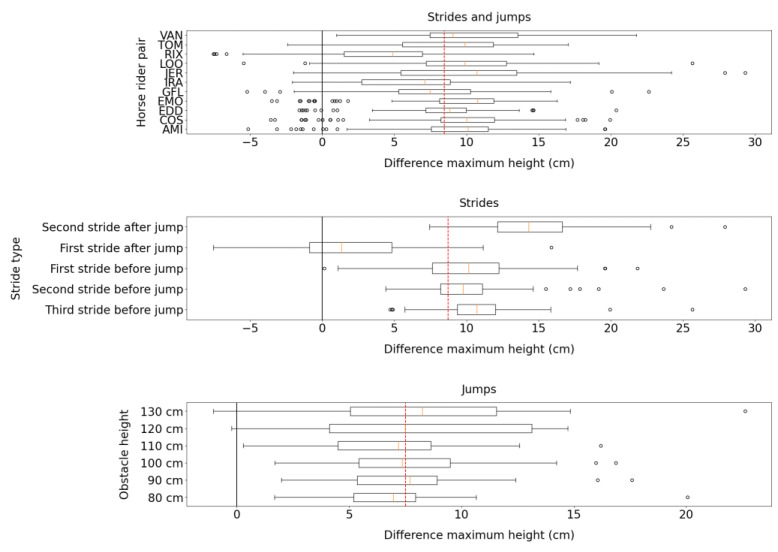
Box plots for the difference in the maximum height (Zmax) measured by the Alogo Move Pro sensor and the OMC system. The red dashed line indicates the mean difference over categories. Box plots show three splits by HRP, stride type (only for strides), and obstacle height (only for jumps).

Figure 4 shows that the Alogo Move Pro sensor was more precise in measuring the maximum height (Zmax) of strides before a jump than strides after a jump. It was less precise in measuring the maximum height (Zmax) of a jump when obstacle height increased.

### 3.3. Stride/Jump Length (Lhorz)

**Figure 5:**
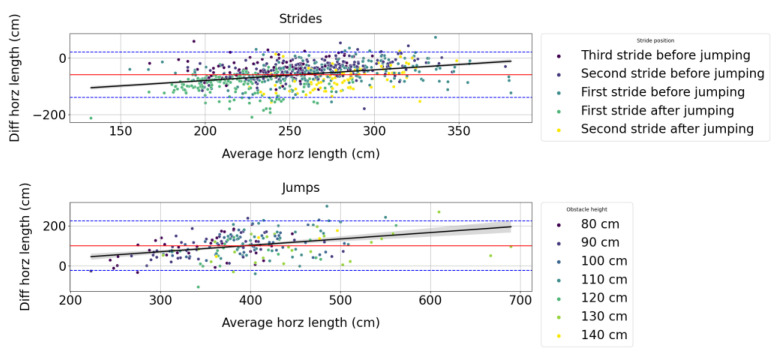
Bland–Altman plots for the stride/jump length (lhorz) measured by the Alogo Move Pro sensor versus the OMC system for strides and jumps. “Diff” contraction stands for difference. The red line indicates the average difference, and the blue dashed lines the 95% limits of agreement. The black line indicates the ordinary least square regression and its 95% confidence interval obtained by a 1000 bootstrap resample.

From Figure 5, we can observe that the difference in measures between the Alogo Move Pro sensor and the OMC system for the stride/jump length variable (lhorz) is positively correlated with the true value.

**Figure 6:**
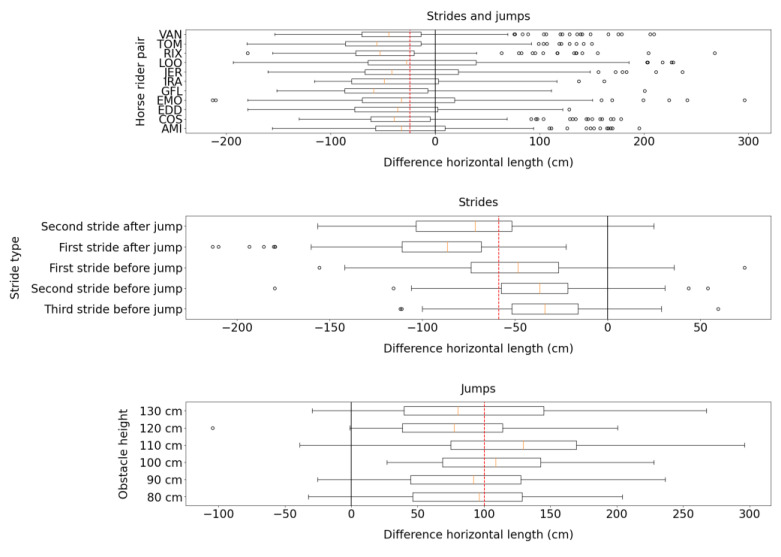
Box plots for the difference in the stride/jump length (lhorz) measured by the Alogo Move Pro sensor and OMC system. The red dashed line indicates the mean difference over categories. Box plots show three splits by HRP, stride type (only for strides), and obstacle height (only for jumps).

Figure 6 shows that the Alogo sensor was more accurate in measuring the length of strides (lhorz) before a jump than strides length after a jump.

### 3.4. Mean Horizontal Speed (Vhorz)

**Figure 7:**
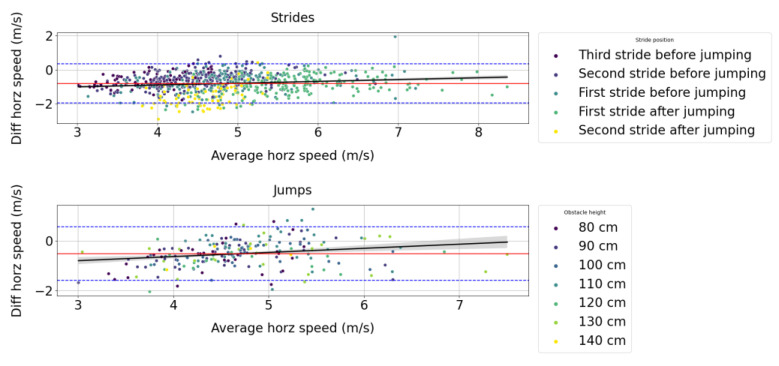
Bland–Altman plots for the mean horizontal speed (vhorz) measured by the Alogo Move Pro sensor versus the OMC system for strides and jumps. “Diff” contraction stands for difference. The red line indicates the average difference, and the blue dashed lines the 95% limits of agreement. The black line indicates the ordinary least square regression and its 95% confidence interval obtained by a 1000 bootstrap resample.

From Figure 7, we can observe that the difference in measures between the Alogo Move Pro sensor and the OMC system for the mean horizontal speed variable (vhorz) positively correlates with the true value.

**Figure 8:**
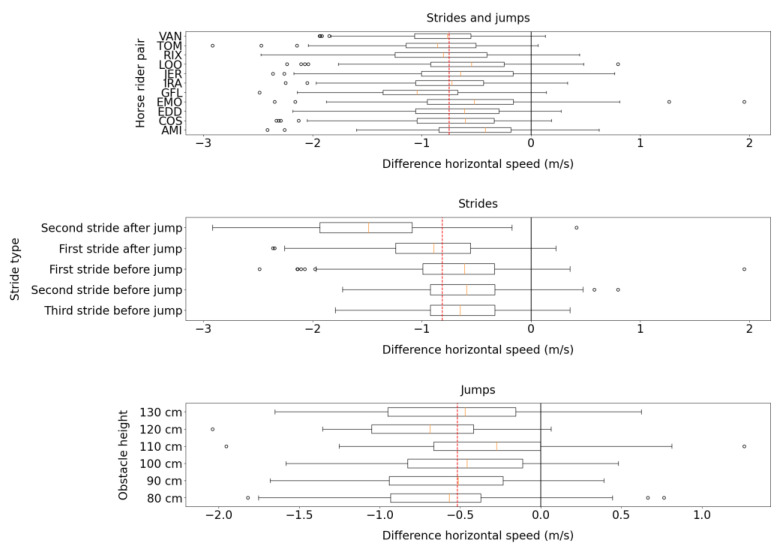
Box plots of the difference of the mean horizontal speed (vhorz) measured by Alogo Move Pro sensor and OMC system. The red dashed line indicates the mean difference over categories. Box plots show three splits by HRP, stride type (only for strides), and obstacle height (only for jumps).

Figure 8 shows that the Alogo sensor was more accurate in measuring the mean horizontal speed variable (vhorz) of strides before a jump than strides after a jump.

## 4. Discussion

The comparison system Qualisys AB used in this study, referred to as the “Gold Standard”, is a very sophisticated, high-value OMC system. It would have been helpful to compare our device using other systems of equivalent technology. As we could not do so, our results and values for certain variables are difficult to compare. The technology used in this device fuses the GPS velocity and the accelerometer data. An IMU is mainly composed of accelerometers and gyroscopes. These sensors have intrinsic biases when measuring quantities, including constant bias, scale factor, thermomechanical white noise, and flicker noise. If the traditional Newton’s mechanics is used, a double integration process is necessary first to estimate velocities from measured accelerations and second to estimate positions from velocities. This would lead to a positional drift proportional to time, associated with an exponential position error propagation. This issue is corrected by adding information from an external reference, such as the GPS data (position and velocity). These observations are fused using the well-known extended Kalman filter (EKF) with different coupling strategies. Because the GPS errors are bounded, the state estimation (position and orientation) using EKF can be very accurate and constant over time [13]. As GPS observations frequency (typically 20 Hz) is lower than IMU update frequency (typically 100 Hz or more), IMU data are used between two observations. As time ranges are short, no positional drift is observed unless the GPS signal is lost too often. 

A previous study by Warner et al. [14] evaluated the agreement between a generic IMU-based system and the OMC system. They also noted variations in the range of motion of up to 25%, particularly in the amplitude of the lower parts of the body. However, using the Bland–Altman agreement parameter analysis represents common ground for similar trends and correlation values.

Another issue to be considered concerns the setup of the OMC system; the cameras placed three meters above the ground may have difficulty with depth perception and the marker placement under the saddle close to the Alogo Move Pro device. This argument was discussed extensively with the OMC system technicians when establishing the study design. They indicated that the totality of the markers could be assimilated into a rigid system that the cameras could correctly detect. 

It is also true that the strap used to attach the sensor under the horse has a brass ring used to attach a piece of leather equipment needed for jumping in many sports horses. It may be argued that this brass ring could affect the functioning of the OMC system, acting as a reflector.

These elements could suggest a less optimal assessment and partly explain the reported variations, highlighting the strength of IMU-based systems, especially for measuring certain gait and jumping variables not easily tracked by OMC capture systems.

The circumstances under which this validation was carried out were quite challenging from a GPS data collection perspective. Performing measurements in a relatively small volume underneath a tent with short movement distances and quick changes in horizontal speed might have resulted in errors not necessarily observed under more ideal circumstances. Some variations could have originated in suboptimal GPS signals or sensor fusion algorithm issues. The variations in stride length before and after the jump and those related to the increase in jump height can be explained by the difference in technology between the two systems. Another element that needs to be considered is that under a tent with a heavy metallic structure, Alogo Move Pro lost the GPS signal. As explained in the discussion, a GPS signal is used to correct the accelerometer signal subject to positional drift. This drift is minimal when a continuous and frequent GPS signal is received. However, GPS signal loss implies a considerable drift which is not corrected until a new reception of the GPS signal. Thus, outliers (extreme values) in some measurements could be explained by this problem.

Horse rider also had a definite effect on our results as the quality of the canter, the length of the stride, and a good approach all determined the quality of the parabola over the fence. Different aspects of the height and width of the jumps required riders to have more control. In our study, the variation in riding levels at the jump was quite significant.

Further tests could be performed to calculate more horse locomotion parameters measured by the Alogo Move Pro device, such as the angle of the horse’s body during takeoff and the striking power of the hindlimbs at takeoff. Similarly, the use of the Pitch (longitudinal balance), Roll (lateral balance), and Yaw (straightness) variables could provide more explanatory results suggesting another form of accuracy and reliability of a device based on IMU technology. At the time this study was conducted, comparison with some of these criteria was not feasible with the gold standard Qualisys system.

One advantage of the Alogo Move Pro device is that it analyses gaits at walk, trot, and canter, which many competing IMU- and GPS-technology-based devices currently on the market do not. Using this device on a circle at lunging is also part of the future perspectives to be tested.

## 5. Conclusions

The agreement and congruence between the OMC and Alogo Move Pro devices were good. Accuracy and precision were deemed suitable for field study conditions. Our results suggest that the Alogo Move Pro device can be a good aid to riders and trainers, providing gait analysis during training and competition sessions. It can also help in the early detection of irregularities or lameness that would require veterinary intervention. It can measure several important parameters of the gait and jump parabola with a good level of accuracy that can detect changes due to performance degradation. We also suggest the use of this device in scientific publications. It is indeed a significant advantage to have data comparable to an OMC but in the field, competition, and training.

## Figures and Tables

**Table 1:** General observations for measured parameters. Accuracy is the mean of the difference between the measure of the Alogo Move Pro sensor and the OMC system. Precision is the standard deviation. CI95% is the 95% confidence interval (mean +/− 1.96 std).

		Accuracy	Precision	CI95%
	Zmax (cm)	8.7 (10.5%)	5.3 (6.3%)	[−1.6; 19.1]
Stride	Lhorz (cm)	−59 (20.7%)	41 (14.5%)	[−139; 22]
	vhorz (m/s)	−0.81 (15.4%)	0.59 (11.2%)	[−1.97; 0.34]
	Zmax (cm)	7.5 (5.5%)	3.8 (2.8%)	[0.1; 15.0]
Jump	Lhorz (cm)	100 (29.2%)	63 (18.2%)	[−22; 223]
	vhorz (m/s)	−0.52 (10.4%)	0.54 (10.9%)	[−1.59; 0.55]

**Table 2:** *K*-samples Anderson–Darling test statistic and *p*-values for the difference measured by the OMC system and Alogo Move Pro sensor. Subsamples were performed based on three splits by HRP, stride type (only for strides), and obstacle height (only for jumps).

Parameter	Variable	Statistic Value	*p*-Value
	Zmax	69.6	<1 × 10^−3^
HRP	lhorz	13.9	<1 × 10^−3^
	vhorz (m/s)	39.7	<1 × 10^−3^
	Zmax (cm)	354.6	<1 × 10^−3^
Stride type	Lhorz (cm)	170.6	<1 × 10^−3^
	vhorz (m/s)	106.6	<1 × 10^−3^
	Zmax (cm)	6.2	<1 × 10^−3^
Obstacle height	Lhorz (cm)	2.7	1.7 × 10^−2^
	vhorz (m/s)	19.3	<1 × 10^−3^

## Data Availability

The data supporting the findings of this study are available on request from the corresponding author.
